# YTRP: a repository for yeast transcriptional regulatory pathways

**DOI:** 10.1093/database/bau014

**Published:** 2014-03-07

**Authors:** Tzu-Hsien Yang, Chung-Ching Wang, Yu-Chao Wang, Wei-Sheng Wu

**Affiliations:** ^1^Department of Electrical Engineering, National Cheng Kung University, Tainan, Taiwan and ^2^Institute of Biomedical Informatics, National Yang-Ming University, Taipei, Taiwan

## Abstract

Regulatory targets of transcription factors (TFs) can be identified by the TF perturbation experiments, which reveal the expression changes owing to the perturbation (deletion or overexpression) of TFs. But the identified targets of a given TF consist of both direct and indirect regulatory targets. It has been shown that most of the TFPE-identified regulatory targets are indirect, indicating that TF-gene regulation is mainly through transcriptional regulatory pathways (TRPs) consisting of intermediate TFs. Without identification of these TRPs, it is not easy to understand how a TF regulates its indirect targets. Because there is no such database depositing the potential TRPs for *Saccharomyces cerevisiae* now, this motivates us to construct the YTRP (Yeast Transcriptional Regulatory Pathway) database. For each TF-gene regulatory pair under different experimental conditions, all possible TRPs in two underlying networks (constructed using experimentally verified TF-gene binding pairs and TF-gene regulatory pairs from the literature) for the specified experimental conditions were automatically enumerated by TRP mining procedures developed from the graph theory. The enumerated TRPs of a TF-gene regulatory pair provide experimentally testable hypotheses for the molecular mechanisms behind a TF and its regulatory target. YTRP is available online at http://cosbi3.ee.ncku.edu.tw/YTRP/. We believe that the TRPs deposited in this database will greatly improve the usefulness of TFPE data for yeast biologists to study the regulatory mechanisms between a TF and its knocked-out targets.

**Database URL**: http://cosbi3.ee.ncku.edu.tw/YTRP/

## Introduction

Cells respond to environmental changes mainly through reorganizing their genomic expressions. This kind of regulation is realized by transcriptional regulatory networks controlled by transcription factors (TFs). Identifying the sophisticated architecture of transcriptional regulatory networks would reveal the fundamental aspects of the mechanisms involved in the cellular adaptation to new environments (1).

The first step toward reconstructing transcriptional regulatory networks is to identify regulatory targets of known TFs. Transcription factor perturbation experiments (TFPEs), a powerful high-throughput experimental procedure, are widely used to achieve this goal (2, 3). By using the genome-wide mRNA expression tiling arrays, the regulatory targets of a TF can be identified from the expression changes between the mutant-type and the wild-type cell lysates owing to the perturbation (deletion or overexpression) of that TF. However, the regulatory targets of a TF identified by this approach contain both direct targets and indirect targets, for which regulation may occur through intermediate TFs. That is, TFPEs can identify the regulatory targets of a TF but cannot distinguish direct targets from indirect ones. This ambiguity in TFPEs can be solved by the information provided by another high-throughput experimental procedure, the chromatin immunoprecipitation (ChIP) experiments (4). This experimental procedure is used to identify interactions between TFs and the promoter regions to which they bind. That is, the binding targets of TFs can be identified by the ChIP experiments. Because these two technologies provide complementary information for gene regulation, integration of these two experimental results will give insight into transcriptional regulatory networks (5).

Intuitive overlapping of the regulatory target genes inferred from TFPEs and the binding targets from the ChIP experiments can extract the direct regulatory targets of TFs. Unfortunately, direct overlap of these two lists of target genes has been shown to be scarce, indicating that most of the identified regulatory targets by TFPEs are indirect (3, 5, 6). These indirect targets are mainly regulated by the TF via transcriptional regulatory pathways (TRPs), consisting of intermediate TFs between the TF and its indirect targets (3, 5, 6). Without the TRP information, TFPEs are of limited use for the biologists because the transcriptional regulatory mechanisms between a TF and its indirect targets are unknown at the molecular level. Thus, providing possible TRPs for the TF-gene regulatory pairs identified by TFPEs is vital.

In this study, we constructed the pioneer database depositing the comprehensive TRP information for TFPE-identified TF-gene regulatory pairs. We retrieved the literature-curated binding and regulatory TF-gene pairs from the YEASTRACT database. Although YEASTRACT provided a graphical tool to visualize the deposited TF-gene pairs as a network, this only plotted the edges between the queried TF list and gene list. The intermediate regulatory pathways were not provided by YEASTRACT. Hence, by applying the developed genome-wide TRP mining procedures on these raw pairs, we extracted the TRPs for specified experimental conditions as potential transcriptional regulatory mechanisms for the given TFPE-inferred TF-gene pairs under different experimental conditions. To provide an easy-to-use and user-friendly searching/browsing interface, we constructed the YTRP (Yeast Transcriptional Regulatory Pathway) database for yeast biologists with these comprehensive TRP information and the accompanying literature supports for the TRPs. This will facilitate biologists to design subsequent network dynamic analysis experiments. YTRP is available online at http://cosbi3.ee.ncku.edu.tw/YTRP/.

## Construction and Contents

### Data collection

TF-gene binding pairs and TF-gene regulatory pairs were used to construct the two underlying networks used for enumerating TRPs. These TF-gene pairs were retrieved from the YEASTRACT database (7, 8), which deposited many TF-gene pairs with different kinds of experimental evidence in the literature (downloaded on 31 December 2013). In total, three different types of data were used in YTRP: (i) 41 013 standard TF-gene binding pairs with experimental evidence (supported by the ChIP-chip, band-shift or footprinting experiments) from 401 publications showing that the TF binds to the promoter region of the target gene; (ii) 168 900 standard TF-gene regulatory pairs with experimental evidence (supported by the TFPEs or detailed gene-by-gene analysis) from 936 publications showing that the perturbation (deletion or overexpression) of that TF affects the target gene’s expression significantly; and (iii) 8805 TF-gene direct regulatory pairs under the nine experimental conditions in total with experimental evidence generated by overlapping the above two types of data. These TF-gene direct regulatory pairs are those pairs with literature supports showing that the TF binds to the promoter region of the target gene and the perturbation of the TF affects the target gene’s expression significantly. These 8805 TF-gene direct regulatory pairs can be downloaded from YTRP. The nine different experimental conditions classified by the YEASTRACT database are cycle/morphology, stress, oxygen availability, unstressed log-phase growth (control), nitrogen source quality/availability, carbon source quality/availability, Ion/metal/phosphate/sulfur/vitamin availability, lipid supplementation and complex industrial media.

### TRP mining procedures and construction of YTRP

**Figure 1.**
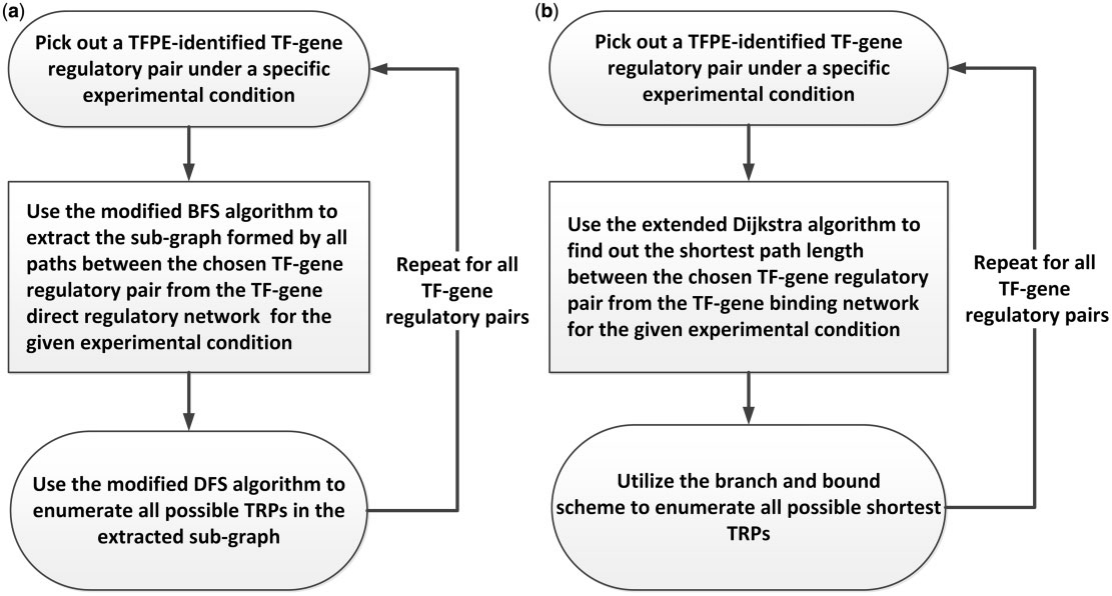
Construction of the YTRP database. The TRP mining procedures for automatically enumerating TRPs from the underlying networks. (**a**) The TRP mining procedure that enumerates all possible TRPs of the given TF-gene regulatory pair from the TF-gene direct regulatory network under different experimental conditions. (**b**) The TRP mining procedure that enumerates all possible shortest TRPs of the given TF-gene regulatory pair from the TF-gene binding network under different experimental conditions.

To get the TRPs, we constructed two transcriptional regulatory networks for each of the nine experimental conditions: the TF-gene direct regulatory network and the TF-gene binding network. Because manual enumeration of TRPs from complex networks requires tremendous amount of time, we mined out the TRP information for the gathered TFPE-identified TF-gene regulatory target pairs under different experimental conditions by TRP mining procedures designed from the graph theory (Figure 1). For the TF-gene direct regulatory network, we used a mining procedure to get all possible TRPs between a given TF and its target gene (Figure 1a). And for the TF-gene binding network, we used a different mining procedure to get all the shortest TRPs between a given TF and its target gene (Figure 1b). All TRPs deposited in YTRP are the data mining results for the two transcriptional regulatory networks under different experimental conditions, and no prediction or selection of TRPs was performed. The construction details are as following.

First, two underlying networks used for enumerating TRPs were constructed for each experimental condition. The first network, called the TF-gene direct regulatory network, was constructed using the TF-gene direct regulatory pairs. A directed edge in the TF-gene direct regulatory network was added when there are curated TF-gene pairs showing that the TF binds to the promoter region of the target gene and the perturbation of the TF affects the target gene’s expression significantly under the specified experimental condition. The second one, called the TF-gene binding network, was constructed using the TF-gene binding pairs. Two nodes connected by a directed edge in the TF-gene binding network represent a TF-gene binding pair with literature supports under the specified experimental condition. Obviously, the constructed TF-gene direct regulatory network is much less complex but with much more biological support than the constructed TF-gene binding network.

Then, for each of the condition-specific TFPE-identified TF-gene regulatory pairs retrieved from the YEASTRACT database, all possible TRPs in the two underlying networks under the specified experimental conditions were enumerated. In the graph theory, it is known that enumerating all possible paths between two nodes in a complex network is an NP-complete problem, which means that no algorithm can solve it in polynomial time when the network is dense (9). Fortunately, because the constructed TF-gene direct regulatory network is sparse, we developed a two-step TRP mining procedure using the divide-and-conquer scheme (10) to achieve this goal efficiently. First, the subgraph formed by all the paths (i.e. TRPs) between the given TF-gene regulatory pair was extracted using the modified breadth-first search algorithm (6, 9). Then we applied the modified depth-first search algorithm (9) to enumerate all possible TRPs in this extracted subgraph. Our two-step procedure can work because the extracted subgraph is much less dense than the original TF-gene direct regulatory network is.

However, this approach could not work when we tried to enumerate all possible TRPs in the TF-gene binding network, which is much more complex than the TF-gene direct regulatory network is. The TF-gene binding network is so dense that extracting the loosely connected subgraph is not possible. In Zhou *et al.*’s work (11), they showed that using the shortest path analysis on the gene expression network can reveal functionally correlated genes. Motivated by their research, we modified our goal to enumerate all possible shortest TRPs for a TF-gene regulatory pair, which can be done with polynomial time complexity (9). We developed a two-step TRP mining procedure to enumerate all shortest TRPs for a given TF-gene regulatory pair in the TF-gene binding network. First, we used the extended Dijkstra’s algorithm in the graph theory (9) to find out the shortest path length between the given TF-gene regulatory pair. Then using this shortest path length as the bound and the DFS algorithm as the search heuristic in the branch and bound searching scheme (10), all the shortest TRPs of the given TF-gene regulatory pair can be enumerated.

### Implementation of the web service of YTRP

YTRP was built using the scripting language Python and PHP. Core network algorithms used in the TRP mining procedures were supported by Networkx, a Python package (12). The network figure containing all possible TRPs for a queried TF-gene pair was generated by Graphviz, an open source graph drawing tool (13).

### Statistics of YTRP

From the YEASTRACT database, 168 900 TF-gene regulatory pairs for 294 TFs were retrieved. Among these, 131 200 TF-gene regulatory pairs are grouped into the nine experimental conditions by YEASTRACT. A TF-gene regulatory pair means that the perturbation of the TF affects the expression of its target gene significantly. However, it is not known whether the TF regulates the target directly or indirectly. Traditionally, biologists only focused on the direct targets and discarded indirect targets because direct targets can be explained simply by overlapping the TFPE data with the ChIP analysis, whereas indirect targets had no easy way to be explained at the molecular level. Unfortunately, only 8805 TF-gene regulatory pairs in total for the nine experimental conditions can be explained by this approach. That is, only 6.7% of the TF-gene regulatory pairs are explainable in direct overlapping of the two data sets. In this database, we tried hard to increase the percentage of the explainable TF-gene regulatory pairs. In the TF-gene direct regulatory network, 10 948 TF-gene regulatory target pairs with TRP information for 149 TFs in total for the nine experimental conditions were found. The pairs are about 8.3% of the TF-gene regulatory pairs. In the TF-gene binding network, 38 090 TF-regulatory target pairs with TRP information for 150 TFs in total for the nine experimental condition can be found. The percentage of the explainable TF-gene regulatory pairs dramatically increased to 29%. The TRP information of each explainable TF-gene regulatory pair provides hypotheses for the molecular mechanisms behind a TF and their regulatory targets, which are worthy of experimental validation by biologists.

For each of the mined-out TRP, we further used a text mining strategy to find the related TRP literature supports for them. We enforced the abstract of the literature to contain all TFs/genes in the queried TRP. According to the curation, we classified the TRPs into known TRPs and putative TRPs. Known TRPs are provided with their related TRP literature supports. The TRP literature information is now deposited in the YTRP database for user referencing. In total, 309 of the 82 549 mined-out TRPs are classified into the category of known TRPs.

## Utility and Discussion

### Database interface

YTRP provides both the search mode and the browse mode for users to query TF-gene pairs of interest. Notice that in YTRP, we aim to provide the TRP information for the TFPE-identified TF-gene regulatory pairs under specified experimental conditions. Hence, only those TF-gene pairs with TFPE information are deposited in the YTRP database. And the TRP information enumerated from the two different underlying networks for the given TF-gene regulatory pair may not coincide because different TRP mining strategies are used for the TF-gene direct regulatory network and TF-gene binding network.

**Figure 2.**
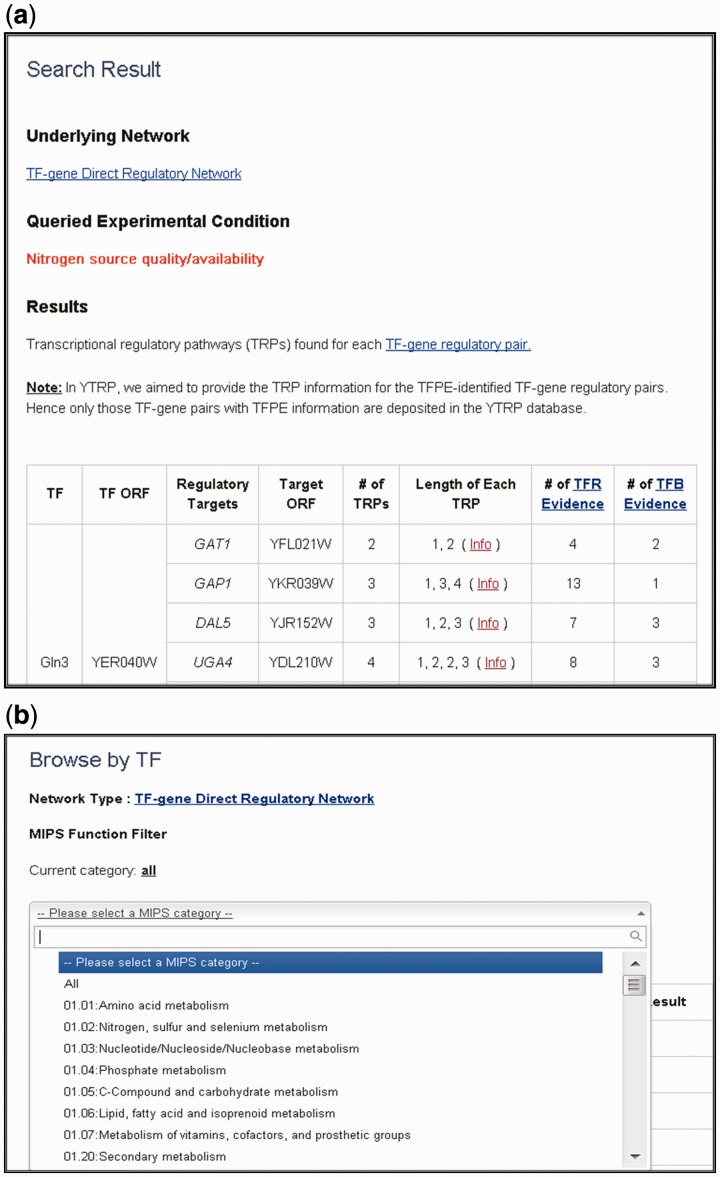
The search result page and the browse page. (**a**) The search results are presented as a table containing all the queried TFPE-identified TF-gene regulatory pairs with their TRP information. TRP information contains the number of enumerated TRPs, the length of each enumerated TRP and the number of experimental evidence for each TF-gene regulatory pair. Clicking the “info” hyperlink directs users to the detail page. (**b**) The browse page contains all regulatory targets of TFs or all regulators of genes with TRP information. In the browse function, users can further filter TFs/genes by their MIPS functional categories.

**Figure 3.**
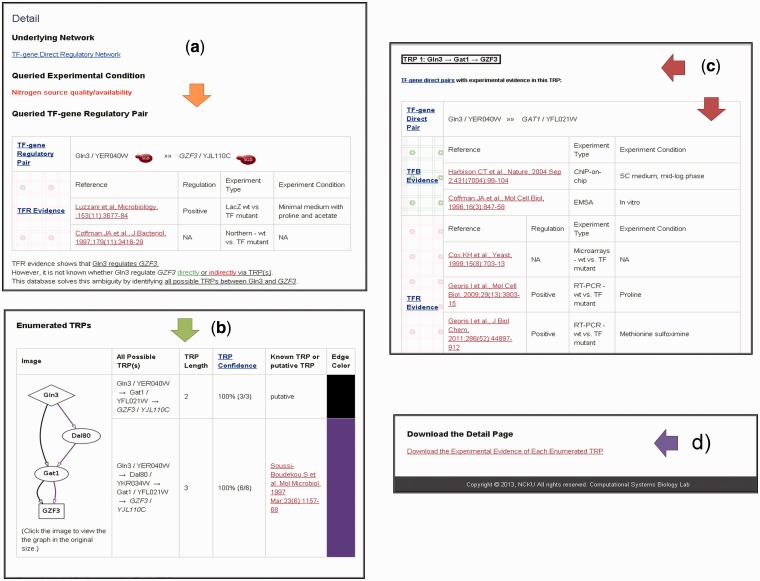
The detail page of the queried TF-gene regulatory pair Gln3-*GZF3*. (**a**) The queried TF-gene regulatory pair and its TFR evidence. (**b**) All enumerated TRPs and their confidence scores. (**c**) The experimental evidence supporting the biological relevance for each enumerated TRP. Only parts of the information are shown because of the space limitations. (**d**) The link that allows users to download the whole detail page.

In the search mode, users can search TRPs for TFPE-identified TF-gene pairs under nine different experimental conditions via three different query ways: (i) user-defined TF list against all genes in the database; (ii) all TFs in the database against user-defined gene list; and (iii) user-defined TF list against user-defined gene list. The search results are presented as a table containing all the TFPE-identified TF-gene regulatory pairs in the input list with mined-out TRP information (Figure 2a). For each TF-gene regulatory pair under the specified experimental condition, YTRP summarizes the number of enumerated TRPs, the length of each enumerated TRP and the number of experimental evidence for each TF-gene regulatory pair. By clicking the hyperlink of a particular TF-gene regulatory pair, users will get a detail page showing a figure containing all possible TRPs for the queried TF-gene regulatory pair under the specified experimental condition (Figure 3). The biological relevance for the edges in the enumerated TRPs is supported by two kinds of experimental evidence: TFR evidence (TF-gene regulation) and TFB (TF-gene binding) evidence. TFR evidence means the experimental evidence in the literature showing that the perturbation (deletion or overexpression) of the TF affects the expression of the target gene significantly. TFB evidence represents the experimental evidence in the literature showing that the TF binds to the promoter region of the target gene. The TRP is classified to be a known TRP if there is TRP literature supports for it.

We use the TF-gene regulatory pair, Gln3-*GZF3* queried under the experimental condition of nitrogen source quality/availability, as an example to demonstrate the contents in the detail page. The detail page consists of four parts. The first part explains why we are interested in the TF-gene pair of Gln3-*GZF3* (Figure 3a) under the queried experimental condition. There exists TFR evidence showing that Gln3 regulates *GZF3* (14). However, it is not known whether Gln3 regulates *GZF3* directly or indirectly. YTRP solves this ambiguity by enumerating all possible TRPs from Gln3 to *GZF3* in the TF-gene direct regulatory network under the experimental condition of nitrogen source quality/availability. The second part shows a figure containing two enumerated TRPs from Gln3 to *GZF3*, which are Gln3→Gat1→*GZF3* and Gln3→Dal80→Gat1→*GZF3* (Figure 3b). Moreover, the confidence score of each TRP is also provided. The confidence score is defined as the proportion of TF-gene pairs with TFR evidence along the direction of the TRP. Take the first TRP (Gln3→Gat1→*GZF3*) as an example. There are three TF-gene pairs along the direction of this TRP: Gln3-*GAT1*, Gat1-*GZF3* and Gln3-*GZF3*. If these three TF-gene pairs all have TFR evidence, i.e. the confidence score equals to 100%, then this TRP is highly experimentally supported and is prone to be biologically true. The rationale is as following. Because Gln3 is in the upstream of the TRP, the perturbation of Gln3 should influence the expression of both *GAT1* and *GZF3*. Similarly, the perturbation of Gat1 should influence the expression of *GZF3*.

The third part lists the experimental evidence of the first TRP (Gln3→Gat1→*GZF3*) (Figure 3c). For Gln3-*GAT1* and Gat1-*GZF3*, they have both TFR and TFB evidence and are called the TF-gene direct regulatory pair. For Gln3-*GZF3*, it has only the TFR evidence and is the TF-gene indirect regulatory target pair. Judging from all the experimental evidence, we are quite confident that the TRP (Gln3→Gat1→*GZF3*) should exist and be functional in yeast cells under the condition of nitrogen source quality/availability. Finally, the fourth part provides a link that allows users to download the whole detail page as a text file (Figure 3d).

In the browse mode, users can browse the YTRP database by TFs. This returns a table listing all direct and indirect regulatory targets of the queried TFs with mined-out TRP information. Users can also browse YTRP by genes. This returns a table listing all the direct and indirect transcriptional regulators of the queried genes with mined-out TRP information. In the table listing all direct and indirect targets of the queried TFs or the table listing all the transcriptional regulators of the queried genes, users can further filter these TFs or genes by their cellular functions classified by the MIPS database (Figure 2b) (15). By clicking the hyperlink of a particular TF-gene regulatory target pairs, there comes out the detail page as illustrated earlier.

### Comparison with related works

The strategies of elucidating the response-to-perturbation mechanisms of genes that are differentially expressed upon knockout of the causative gene are mainly through the integration of different high-throughput assays, such as the protein–DNA binding data or the physical protein–protein interaction data (16). The YTRP database aims to provide all possible potential TRPs between TFs and their indirect regulatory targets inferred from TFPEs in yeast cells. Similar works that provided the TRP information for TFPE data could be roughly dividing into two different types, depending on the adopted mining algorithms and constrains: the optimization method and the path enumeration method.

The first type of method was based on the forming of optimization problems on the constructed regulatory and/or metabolic networks (17–20). This type of algorithm mined out the TRPs for the given TFPE-inferred indirect regulatory pairs based on the arithmetical modeling of the confidence of the TF-gene binding pairs or the protein–protein interaction pairs. TRPs were deduced on the sub-network that satisfies these optimization problems. Research on these algorithms tried to explain as many indirect TF-gene regulatory pairs as possible. But often they suffer from two obstacles. First, only limited and prejudiced TRPs were reported by these algorithms because of the algorithm-defined confidence score (16). This could elucidate few confident transcriptional mechanisms. Further understanding and analysis of the network dynamics cannot be performed. Moreover, solving these optimization problems is often NP-complete or NP-hard, meaning that the algorithms are computationally expensive (16). Genome-wide TF analysis by these algorithms is not possible under current computer architectures (21).

Because of the computational complexity, the second type of method used a different criterion from optimization. They enumerated TRPs from the constructed network with some constrains on the TRP length between the TFPE-inferred TF-gene indirect pairs. Some considered only the TRPs of length two (5, 22). TRPs with path length more than two were considered redundant or unachievable. Others tried to enumerate only one shortest TRP between the TF-gene indirect regulatory pairs (11). These methods all limit potential downstream regulatory dynamic analysis of the reported TRPs.

YTRP specifically aims to detour these obstacles and extract possible TRPs between TFs and their indirect regulatory targets in yeast cells. YTRP provides an advance on the insight of deposited data compared with the original YEASTRACT database (Table 1). Although YEASTRACT provides a graphical tool to visualize the deposited TF-gene pairs as a network, this only plotted the edges between the queried TF list and gene list. This only provides the TRPs with length one. The intermediate regulatory pathways are not provided by YEASTRACT. In contrast, YTRP serves to provide all possible TRPs or all shortest TRPs that are not limited to path lengths or the number of TRPs. Further, we also specified known TRPs and putative TRPs according to the published TRP literature. The developed genome-wide TRP mining procedures are computationally available to elucidate the regulatory mechanisms for genome-wide TFPEs. In addition, YTRP does not have prejudice in choosing TRPs because all mined-out TRPs in a given underlying network are provided for the user and a confident score is provided only for user referencing.

**Table 1.** Major differences between the functionality of YTRP and YEASTRACT

Functional Differences	YEASTRACT	YTRP
Major purpose	To deposit the direct and indirect TF-gene pairs	To provide TRP information for TFPE-inferred TF-gene regulatory pairs
TF-gene regulatory mechanism	Only TRPs with length one	All possible TRPs enumerated from the two underlying networks
Graphical tools	Only plots the input TF list against the input gene list	Generates plots of all possible TRPs for TF-gene regulatory pairs
TRP information	None	Provides known TRP-related literature and deposits experimental evidence for each TF-gene pair of the TRPs

In summary, YTRP serves as a database that provides comprehensive TRP information extracted from constructed underlying transcription regulatory networks. The TRPs mined out by our procedures are not limited to path lengths or confidence restrictions of the searching algorithms. Notice that all TRPs deposited in YTRP are the results of graph data mining on the data provided by YEASTRACT, and no computational prediction or confidence selection was applied to the TRPs. Users can browse YTRP to find the possible TRPs of their interest and choose the TRP hypotheses provided by YTRP for further analysis on their own expert knowledge.

### Issues related to YTRP

YTRP deposits comprehensive TRP information for elucidating the transcriptional regulatory mechanisms for the TF-gene indirect regulatory pairs inferred from TFPEs. We mined out the TRP information using a network data mining approach on the integration of TF-binding data and the TFPE data. Yet these TRP mining procedures used by YTRP inherit the potential drawback of systems biology. In systems biology, a cellular system is perturbed and measured by the high-throughput technologies. Thus, an understanding of the system is based on the integration of different high-throughput assays (23). But this type of data integration often suffers the system bias caused by the fact that different high-throughput data were performed on different cell states and experimental conditions. In addition, data-depositing databases such as YEASTRACT often mix data from different cell states, different strains and different experimental conditions (24). Recent research tried to solve the system bias by adjusting the *P*-value threshold of different integrated high-throughput data (25), but this type of systematic bias is still unavoidable.

In YTRP, we deposited all the comprehensive TRP information using the TRP mining procedures. These TRPs are much more manageable than the original dense transcription regulatory network. Hence, this inevitable systematic bias inherited from the approach of data integration can be reduced by expert knowledge or suitable downstream expression analysis. Furthermore, the dynamic of activation/repression wiring of these TRPs also requires further condition-specific gene expression data (26). The major purpose of YTRP is to provide an easy-to-use interface for biologists to browse these potential TRPs and to facilitate the downstream regulatory dynamic analysis. We merely provide the TRPs with regulatory confidence for referencing and leave the interpretation freedom to the users for more flexible application of this database.

### Case study

**Figure 4.**
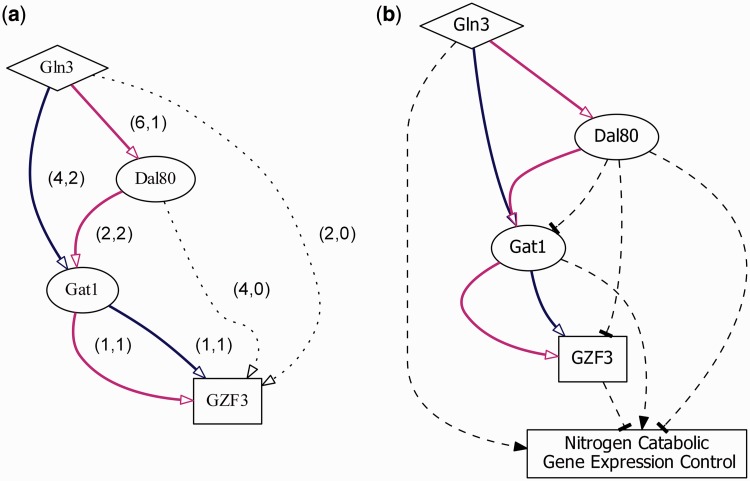
Nitrogen catabolic gene expression control. Two enumerated TRPs of the TF-gene regulatory pair: Gln3-*GZF3*. (**a**) In the figure, (x, y) stands for the number of TFR and TFB evidence, respectively, that support the biological relevance for each TF-gene pair along the direction of the two enumerated TRPs (Gln3→Gat1→*GZF3* and Gln3→Dal80→Gat1→*GZF3*). Dotted lines indicate indirect regulation. (**b**) Transcriptional regulatory dynamics inferred from the condition-specific expression data. Dashed lines represent activation (arrowhead) or repression (tee head) regulation.

We give a case study showing the application and utilization of YTRP. *Saccharomyces cerevisiae* can use a wide variety of nitrogen sources. The expression of nitrogen catabolic genes is controlled by the action of four GATA family transcription factors: Gln3, Gat1, Dal80 and Gzf3 (27). The first two TFs act as the positive regulators and the last two function as the negative regulators. It is known that the perturbation of Gln3 causes the expression of *GZF3* to change significantly under the experimental condition of nitrogen source quality/availability, but Gln3 does not bind to the promoter region of *GZF3* (14, 28). That is, *GZF3* is an indirect regulatory target of Gln3. Without TRP information, it is hard to understand how Gln3 regulates *GZF3*. Querying the TF-gene regulatory pair Gln3-*GZF3* in YTRP on the TF-gene direct regulatory network as the underlying network under the experimental condition of nitrogen source quality/availability returns two TRPs: Gln3→Gat1→*GZF3* and Gln3→Dal80→Gat1→*GZF3* (Figure 4a). These two TRPs are fully experimentally supported because both of their confidence scores are 100%, meaning that there exists TFR evidence for every TF-gene pair along the direction of the TRPs.

Taking the second TRP (Gln3→Dal80→Gat1→*GZF3*) as an example, we can find various experimental supports from the literature showing that (i) Gln3 directly binds (to the promoter region of *DAL80*) and regulates *DAL80* and indirectly regulates *GAT1* and *GZF3*; (ii) Dal80 directly binds (to the promoter region of *GAT1*) and regulates *GAT1* and indirectly regulates *GZF3*; and (iii) Gat1 directly binds (to the promoter region of *GZF3*) and regulates *GZF3*. From the subsequent condition-specific mRNA expression analysis, it is further shown that Dal80 represses *GAT1* and *GZF3* (27, 28) (Figure 4b). These two identified TRPs are experimentally confirmed and can be found in the transcriptional regulatory model of nitrogen catabolic genes, which was established after the experimental efforts of numerous research groups (1).

## Conclusions

In this article, we present the YTRP database that provides comprehensive TRP information for TF-gene regulatory pairs inferred from TFPEs. The enumerated TRPs provide experimentally testable hypotheses of the molecular mechanisms behind TFs and their regulatory targets. We believe that the TRPs deposited in YTRP will greatly improve the usefulness of TFPE data for yeast biologists to study gene transcriptional regulation. YTRP has an easy-to-use interface for biologists to search and browse for TRP information of the TF-gene regulatory pairs of interest. YTRP will be regularly updated based on the newly published literature on TRPs and the latest release of the YEASTRACT database.

## Funding

This work was funded by Taiwan National Science Council [NSC 099-2628-B-006-015-MY3]. And the funding of open access charge was supported by Taiwan National Science Council [NSC 102-2218-E-010-005-MY2].

*Conflict of interest*. None declared.
